# Unveiling Mechanistic Complexity in Manganese-Catalyzed C–H Bond Functionalization Using IR Spectroscopy Over 16 Orders of Magnitude in Time

**DOI:** 10.1021/acs.accounts.3c00774

**Published:** 2024-02-27

**Authors:** Ian J. S. Fairlamb, Jason M. Lynam

**Affiliations:** Department of Chemistry, University of York, Heslington, York YO10 5DD, United Kingdom

## Abstract

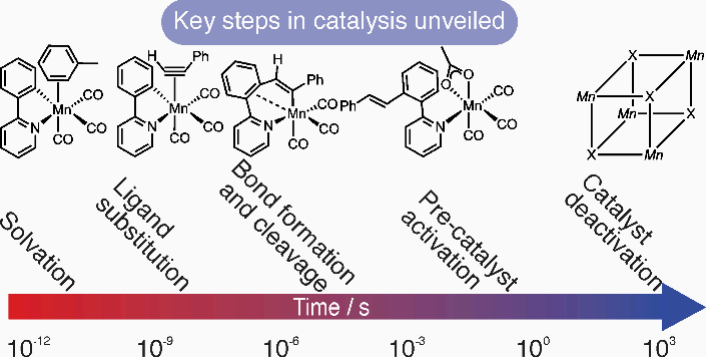

An understanding of the mechanistic processes that underpin reactions catalyzed by 3d transition metals is vital for their development as potential replacements for scarce platinum group metals. However, this is a significant challenge because of the tendency of 3d metals to undergo mechanistically diverse pathways when compared with their heavier congeners, often as a consequence of one-electron transfer reactions and/or intrinsically weaker metal–ligand bonds. We have developed and implemented a new methodology to illuminate the pathways that underpin C–H bond functionalization pathways in reactions catalyzed by Mn–carbonyl compounds. By integrating measurements performed on catalytic reactions with in situ reaction monitoring and state-of-the-art ultrafast spectroscopic methods, unique insight into the mode of action and fate of the catalyst have been obtained.

Using a combination of time-resolved spectroscopy and in situ low-temperature NMR studies, we have shown that photolysis of manganese–carbonyl precatalysts results in rapid (<5 ps) CO dissociation—the same process that occurs under thermal catalytic conditions. This enabled the detection of the key states relevant to catalysis, including solvent and alkyne complexes and their resulting transformation into manganacycles, which results from a migratory insertion reaction into the Mn–C bond. By systematic variation of the substrates (many of which are real-world structurally diverse substrates and not simple benchmark systems) and quantification of the resulting rate constants for the insertion step, a universal model for this migratory insertion process has been developed. The time-resolved spectroscopic method gave insight into fundamental mechanistic pathways underpinning other aspects of modern synthetic chemistry. The most notable was the first direct experimental observation of the concerted metalation deprotonation (CMD) mechanism through which carboxylate groups are able to mediate C–H bond activation at a metal center. This step underpins a host of important synthetic applications. This study demonstrated how the time-resolved multiple probe spectroscopy (TR^M^PS) method enables the observation of mechanistic process occurring on time scales from several picoseconds through to μs in a single experiment, thereby allowing the sequential observation of solvation, ligand substitution, migratory insertion, and ultimate protonation of a Mn–C bond.

These studies have been complemented by an investigation of the “in reaction flask” catalyst behavior, which has provided additional insight into new pathways for precatalyst activation, including evidence that alkyne C–H bond activation may occur before heterocycle activation. Crucial insight into the fate of the catalyst species showed that excess water played a key role in deactivation to give higher-order hydroxyl-bridged manganese carbonyl clusters, which were independently found to be inactive. Traditional in situ IR and NMR spectroscopic analysis on the second time scale bridges the gap to the analysis of real catalytic reaction systems. As a whole, this work has provided unprecedented insight into the processes underpinning manganese-catalyzed reactions spanning 16 orders of magnitude in time.

## 1 Key References

- YahayaN. P.; ApplebyK. M.; TehM.; WagnerC.; TroschkeE.; BrayJ. T. W.; DuckettS. B.; HammarbackL. A.; WardJ. S.; MilaniJ.; PridmoreN. E.; WhitwoodA. C.; LynamJ. M.; FairlambI. J. S.Manganese(I)-Catalyzed C–H Activation: The Key Role of a 7-Membered Manganacycle in H-Transfer and Reductive Elimination. Angew. Chem. Int. Ed.; 2016, 55, 12455–12459.10.1002/anie.201606236PMC511368027603008(1) This work established that seven-membered manganacycles play a key role as branching points to different products in Mn-catalyzed C–H bond functionalization reactions.
- HammarbackL. A.; ClarkI. P.; SazanovichI. V.; TowrieM.; RobinsonA.; ClarkeF.; MeyerS.; FairlambI. J. S.; LynamJ. M.Mapping out the Key Carbon–Carbon Bond-Forming Steps in Mn-Catalyzed C–H Functionalization. Nat. Catal.; 2018, 1, 830–840.(2) The direct observation of catalytically relevant carbon–carbon bond formation with the coordination sphere of manganese is reported through the use of time-resolved spectroscopic methods.
- HammarbackL. A.; RobinsonA.; LynamJ. M.; FairlambI. J. S.Mechanistic Insight into Catalytic Redox-Neutral C–H Bond Activation Involving Manganese(I) Carbonyls: Catalyst Activation, Turnover, and Deactivation Pathways Reveal an Intricate Network of Steps. J. Am. Chem. Soc.; 2019, 141, 2316–2328.30698423 10.1021/jacs.8b09095(3) The key pathways leading to catalyst activation and deactivation for Mn–carbonyl catalysts are reported, including how the interaction with water leads to the formation of inactive Mn clusters.
- HammarbackL. A.; AucottB. J.; BrayJ. T. W.; ClarkI. P.; TowrieM.; RobinsonA.; FairlambI. J. S.; LynamJ. M.Direct Observation of the Microscopic Reverse of the Ubiquitous Concerted Metalation Deprotonation Step in C–H Bond Activation Catalysis. J. Am. Chem. Soc.; 2021, 143, 1356–1364.33428402 10.1021/jacs.0c10409(4) Time-resolved spectroscopy enables the direct observation of intramolecular protonation of an Mn–C bond by carboxylic acid. A multistep process involving solvation, ligand substitution, C–C bond formation, and Mn–C bond protonation is observed in a single experiment.

## 2 Introduction

An understanding of the mechanistic processes that underpin transition-metal-catalyzed reactions is vital for the discovery and development of highly active and selective catalysts for applied synthetic chemistry. Moreover, scalability and reproducibility often depend on understanding the impact of reaction sensitivities relating to mechanistic implications and consequences. This is especially important in the development of catalyst systems based on Earth-abundant 3d transition metals because they are potential replacements for systems based on scarcer, supply-sensitive, and valuable platinum group metals. However, diverse mechanistic manifolds are available for these elements because 3d metal complexes have weaker metal–ligand bonds and have a propensity for one-electron, rather than two-electron, transfer reactions when compared with 4d and 5d cogeners.^5,6^

**Figure 1:**
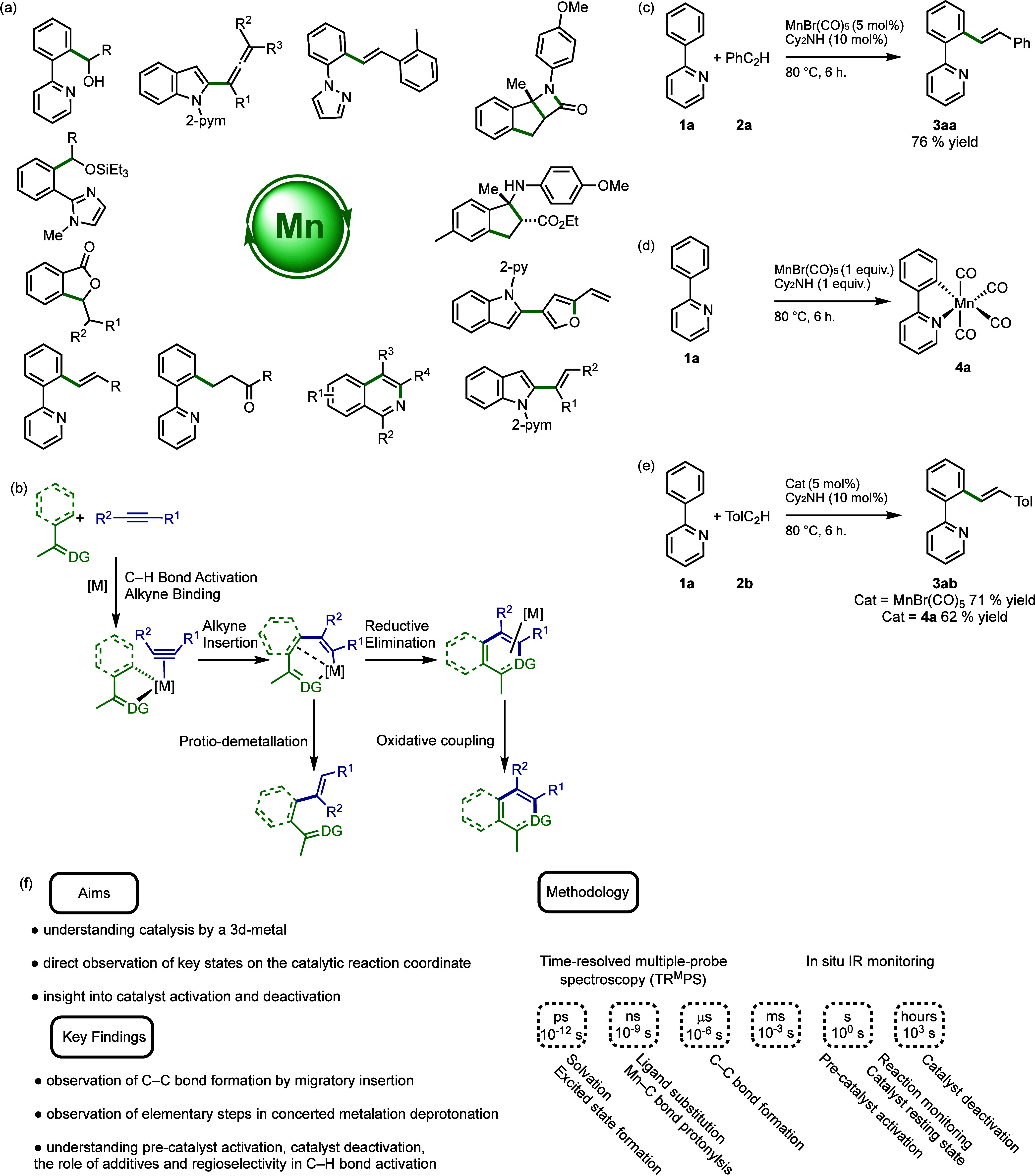
(a) An example of the products formed in Mn-catalyzed reactions. The bonds formed in the Mn-catalyzed step are highlighted in green. (b) Key steps in the Mn-catalyzed reactions (DG = directing group). (c) Coupling of 2-phenylpyridine, **1a**, with phenylacetylene, **2a**. (d) Formation of **4a** from **1a** in the presence of base. (e) Control experiments to show **4a** is a viable catalyst.^13^ (f) Key findings from this work and methods used.

One of the most important developments in 3d metal catalysis is in C–H bond functionalization reaction processes. These transformations have traditionally been the purview of platinum-group-based catalysts. The recent development of manganese-^7−11^ and cobalt-catalyzed^12^ C–H bond functionalization reactions represents a significant synthetic advance, mainly as different reaction pathways and products result. A remarkable facet of manganese-catalyzed reactions is relatively simple precatalysts, e.g., MnBr(CO)_5_ and Mn_2_(CO)_10_, are able to perform C–H bond functionalization reactions between an eclectic array of coupling partners giving access to a diverse range of products (Figure 1).^7−11^ Central to the success of this strategy was the initial pairing by Wang and co-workers of MnBr(CO)_5_ with an amine base; this enabled the coupling of heterocycles, e.g., **1a** (Figure 1c), with alkynes, **2a**, to give alkenylated products, **3aa**.^13^ Stoichiometric studies demonstrated that the interplay between MnBr(CO)_5_, **1a**, and the amine base gave manganacycles [Mn(C∧N)(CO)_4_], **4** (Figure 1d). These compounds were initially prepared by Bruce and co-workers from MnBn(CO)_5_ and an appropriate heteroaromatic compound, such as **1a**.^14^ Importantly in the catalytic work, compounds such as **4a** are catalytically competent (Figure 1e). Despite this, additional advances are needed to translate these species to be genuine replacements for platinum group metals (PGMs)—a true test is seeing their application in target-orientated synthesis. These include (1) reducing the catalyst loading, which is typically 10 mol % in manganese, (2) ensuring that there is a wide functional group tolerance to improve the applicability of the chemistry, and (3) development of the next generation of Mn catalysts in which the coligands can influence the outcome of the reaction by tuning the steric and electronic properties of the metal beyond what is capable with CO. For example, opportunities exist to exploit other unsaturated small molecules, as has been achieved with the PGMs, and to discover new transformations.

To achieve these goals, a comprehensive understanding of the mechanistic processes that underpin the precatalyst activation, the steps constituting the catalytic reaction coordination, and the fate of the catalyst are needed (Figure 1f). In this Account, we describe our studies that have provided insight into all these aspects relating to Mn-catalyzed C–H bond functionalization reactions and how exploring both the fastest and slowest steps underpinning the reactions is important in understanding catalysis more holistically.

## 3 Techniques and Methodology

Gaining insight into the mechanism of a catalytic reaction can be performed in several ways. The kinetic profile of the reaction obtained by monitoring the change in conversion of starting material and product concentrations can provide key information about rate-determining states and off-cycle species and side reactions. Modern analysis methods, such as variable time normalization analysis (VTNA) and reaction progress kinetic analysis (RPKA), have proven invaluable.^15^ Complementing these experiments with in operando reaction monitoring provides a convenient way to explore catalyst speciation under the reaction conditions. In the case of reactions catalyzed by Mn–carbonyl complexes, IR spectroscopy provides insight into catalyst speciation because the position, intensity, and dynamics of the bands in the spectrum due to the vibrational modes of these ligands are effectively reporters for changes in the coordination environment of the metal.^16^ Furthermore, the short time scale of the IR experiment entails that chemical processes on picosecond to hour time scales can potentially be investigated with appropriate instrumentation. This approach provides detailed insight into the pathways underpinning precatalyst activation and subsequent catalyst deactivation. Long-lived catalytic intermediates or off-cycle species can also be observed.

It is important to mention that species that are on the catalytic reaction coordinate are short-lived by definition and present in low concentration, which makes their direct observation difficult, if not impossible, in some systems. Metal–carbonyl complexes offer a way to circumvent these problems as, in addition to their strong and sensitive reporting groups in the IR spectrum, CO loss from metal carbonyls may be stimulated photochemically.^16^ When CO loss is a key step in thermal catalyst activation, then it is possible to use light to simulate the same chemical process. Therefore, time-resolved spectroscopy can be used to explore the behavior of Mn–carbonyl-based catalysts.

Time-resolved experiments operate on the principle of pump–probe spectroscopy. Here the pump activates the system generating a nonequilibrium excited state. The probe, which occurs at a later time (i.e., the pump–probe delay), then examines the nature of the system as it returns to equilibrium. In the case of the studies discussed herein, time-resolved infrared spectroscopy (TRIR) is used to explore Mn catalyst behavior. The pump is an ultrafast UV laser pulse, which expels a CO ligand from a metallacycle, such as **4a**. The fate of the light-activated complex is then monitored by a subsequent probe pulse in the mid-IR with the position and intensity of vibrational modes of the retained CO ligands providing information about the speciation and dynamics of the resulting photoproducts. Fundamentally this light-induced CO loss mirrors the process that occurs thermally, which ensures that the results relate to the catalytic reaction coordinate. The TRIR experiments take advantage of the time-resolved multiple probe spectroscopy^17^ (TR^M^PS) technique at the ULTRA facility (Rutherford Appleton Laboratory).^18^ By synchronizing the pump and probe laser pulses with a combination of optical and electronic delays, TR^M^PS enables light-induced events to be observed on time scales from 0.5 ps through to 1 ms in a single experiment. This temporal flexibility enables the observation and quantification of different catalytic events. These experiments take advantage of the inert nature of the manganese complexes, such as **4a**: in the absence of light at room temperature, the complexes are robust and can be dissolved, unchanged, in a range of media, thereby ensuring that light can be used to trigger the desired chemistry.

Sections 4–8 describes our results, which have used both in operando IR spectroscopy and the TR^M^PS method, to explore the mechanistic processes that underpin Mn catalysis methods. These two methods give complementary mechanistic information covering time scales ranging from picoseconds through to hours, which provides hitherto unprecedented insight into the bond formation and activation processes that underpin catalysis.

## 4 Direct Observation of Carbon–Carbon Bond Formation by Migratory Insertion of Alkynes into a Mn–C Bond

The initial mechanistic hypothesis presented by Wang and co-workers for the alkenylation of 2-phenylpyridine involves the formation of complexes **4a** through a base-assisted C–H bond activation (Figure 1d).^13^ Subsequent CO loss then permitted alkyne binding and migratory insertion. The resulting metallacycle, **5**, is proposed to be a key state in the whole catalytic process as protonation of the newly formed Mn–C bond would then yield the alkenylation product, whereas reductive elimination from this species provides a route to annulated products (Figure 1b).

**Figure 2:**
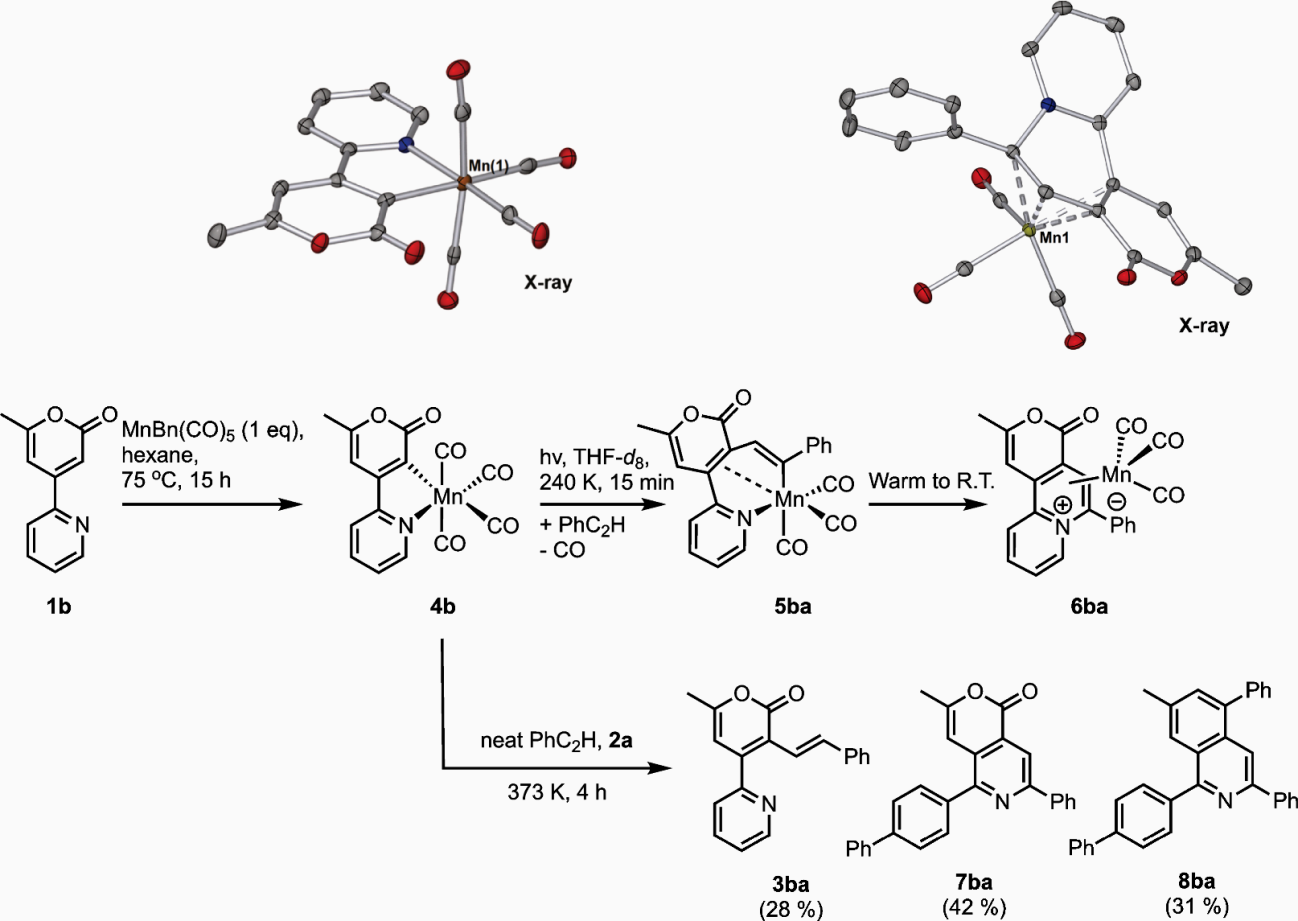
Synthesis of complex **4b** and subsequent thermal and photochemical reactions.

Our initial studies probed the mechanistic steps that lead to metallacycle, **5**, by using pyrone-substituted complex **4b** (a model reaction substrate).^1^ Low-temperature photolysis of **4b** in the presence of PhC_2_H, **2a**, allowed complex **5ba** (Figure 2) to be identified by NMR spectroscopy. The formation of **5ba** corresponds to the migratory insertion of the alkyne into a Mn–C bond of **4b**. Observation of **5ba** identified a reaction anvil point. In the absence of an alkyne, reductive elimination gives **6ba**; however, reaction of **4b** with neat phenylacetylene affords the alkenylation product, **3ba**, as well as **7ba** and **8ba** arising from cycloaddition reactions.

The observation of **5ba** highlights a critical point for a mechanistic study. Specifically, several microscopic steps need to occur (loss of a CO–ligand, coordination of the alkyne, and then the migratory insertion step) to form the C–C and Mn–C bonds in **5ba**. These steps are very fast because they could not be observed, for example, by low-temperature NMR spectroscopy. Therefore, TR^M^PS was used to investigate these hitherto hidden steps because it provides a method to observe events occurring on time scales as short as 0.5 ps.

**Figure 3:**
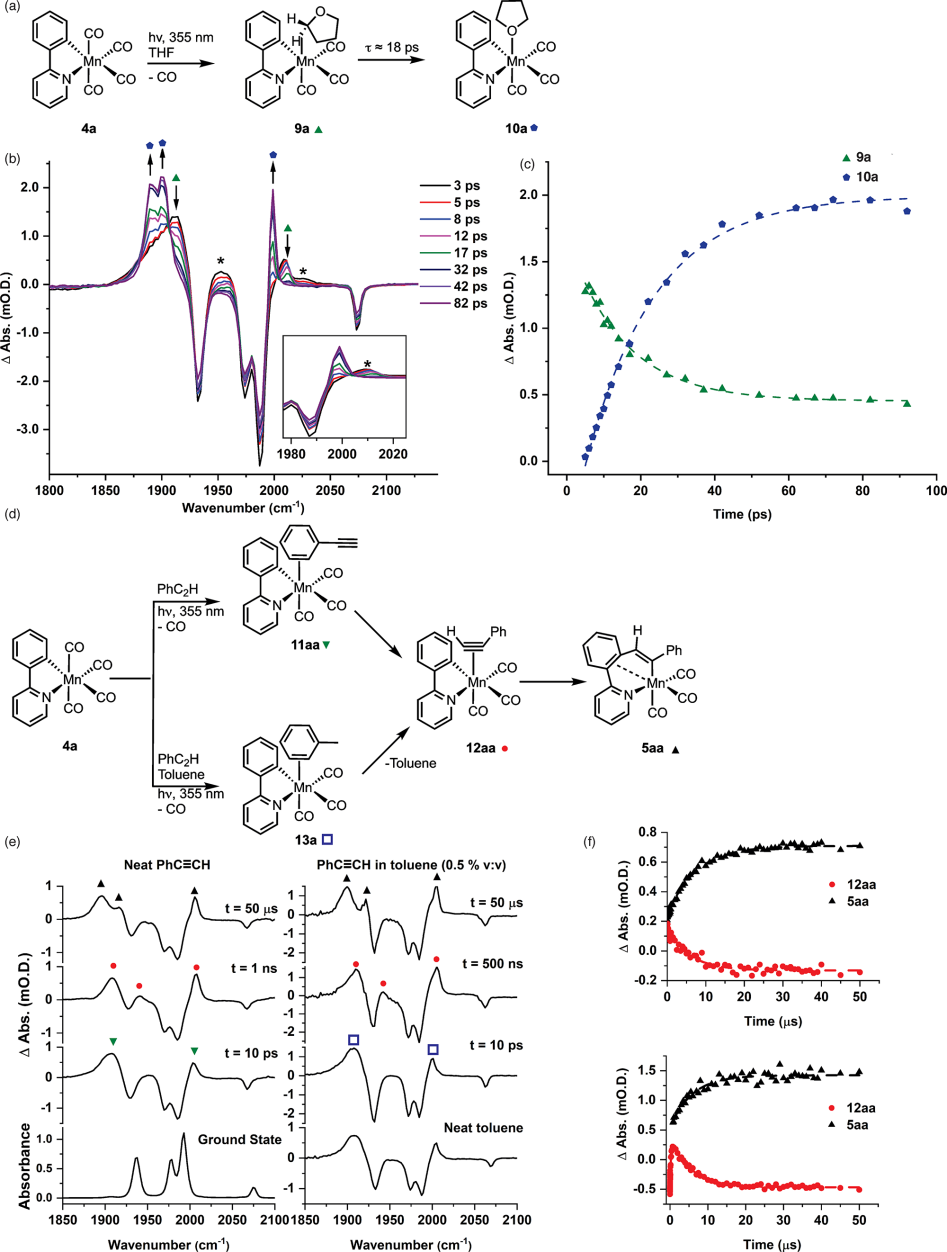
(a) Light-induced loss of CO from **4a** in THF solution. (b) Difference spectra and (c) kinetic profiles showing the conversion of **9a** into **10a**. (d) Reaction scheme showing the product formed from the photolysis of **4a** in the presence of PhC_2_H. (e) Difference spectra and (f) kinetic profiles showing the formation of **12aa** and conversion to **5aa**.

Using the archetypical manganacycle **4a** as an example, irradiation results in the loss of a carbonyl ligand in less than 5 ps and formation of the solvent (S) complexes *fac*-[Mn(ppy)(CO)_3_(S)].^19^ A small amount of a second species with a short (∼4 ps) lifetime was observed, which was assigned to the triplet excited state, ^**3**^**4a**. The solvent complexes are formed in vibrationally excited states, and cooling to the ν = 0 state occurs in <50 ps. This initial binding event is kinetically controlled. The solvent is the most abundant species present in the experiments and, therefore, binds in preference to any solutes. However, the binding to the solvent itself is also a kinetically controlled event. For example, in THF, the TRIR spectra indicate that the Mn binds initially to the cyclic ether C–H bonds (**9a**, Figure 3a) prior to isomerization to the thermodynamically preferred O-binding mode, **10a**, (which is how one would depict it as an undergraduate chemist, i.e., the lowest energy arrangement). Evidence for this is provided by the band positions in the different spectra (Figure 3b) at short pump–probe delays, which are very close to those observed when the experiment is performed in heptane solution (where binding of the solvent is only possible through a C–H bond). Over the course of ∼18 ps, the formation of new IR bands at lower energy in the spectra is consistent with the binding of a better donor (the oxygen atom) to the metal. Moreover, experiments performed in di-*n*-butyl ether demonstrate that the lifetime of the C–H-bonded species (to Mn) is longer, which is consistent with stochastic chain-hopping to form the most preferred bonding mode. Related observations are present across a range of different solvent systems and presumably represent an ultrafast solvent binding event, which is governed by the topology of the first solvation sphere of the complex.

Detailed information about the bond-forming events that underpin Mn-catalyzed C–H bond functionalization experiments was obtained by examining the behavior of the light-activated complexes in the presence of reaction substrates. For example, photolysis of a PhC_2_H solution of **4a** results in kinetically controlled binding of the alkyne to the metal through the arene to give **11aa** (Figure 3d).^2^ A rearrangement to the thermodynamically more stable π-bound form, **12aa**, then occurs in ∼50 ps. Insertion of the alkyne into the Mn–C bond of the manganacycle occurs to give **5aa** over the course of ∼20 μs. Repeating the experiment in a toluene solution of **1a** and PhC_2_H resulted in a related series of observations. First, initial binding of the metal to the toluene solvent occurred to give **13a**, followed by alkyne substitution to give the previously observed π-bound alkyne complex **12aa**. A pseudo-first-order kinetic analysis demonstrated that the solvent substitution was second order with a rate constant of (3.74 ± 0.16) × 10^7^ mol^–1^ dm^3^ s^–1^.^20^ Migratory insertion of the alkyne into the Mn–C bond was zero order in alkyne and first order in Mn complex, with a first-order rate constant of (1.43 ± 0.03) × 10^5^ s^–1^, which is statistically identical to that observed for the reaction performed in neat alkyne (1.35 ± 0.09) × 10^5^ s^–1^, thereby confirming the intramolecular nature of this process.

With this methodology established, it was possible to quantify the effects of changing the alkyne and the manganacycle on the rate constant for the migratory insertion reaction.^20−22^ These experiments demonstrated that electron-withdrawing groups on the alkyne accelerated the rate of the reaction when compared with donating groups. From a steric perspective, alkynes with bulky substituents (including disubstituted alkynes) retarded the rate. Although the effects of changing the steric^23−25^ and electronic^26−30^ parameter of alkynes on migratory insertion reactions have been previously observed, this approach allowed for the microscopic rate constants for alkyne insertion to be directly measured. This permitted a global computational model for this process to be developed and correlated with the experimental results.^20^ The key finding from the computational analysis was that the rate of the MI reaction could be correlated to a synergic bonding interaction between the nascent C–C bond that is formed in this step and metal-based d-orbitals. This was found to be enhanced by electron-withdrawing alkynes and retarded in those with electron-donating groups or those with sterically demanding substituents. Although this could be related to the changes in orbital energies with the change in substituent, it may also be a function of the metal–alkyne distance, which will be shorter with electron-withdrawing alkynes (because of enhanced π-backdonation), hence facilitating synergic back-bonding between the nascent C–C bond and the metal. The opposite is true for electron-rich alkynes or those with bulky groups. This model also provides an explanation for the regiochemistry of alkyne insertion. Here, the less favored 1,2-insertion will involve the formation of a bond to the carbon atom of the alkyne bearing the bulkier substituent. Consequently, this carbon atom will be more remote from the metal, which minimizes the back-bonding from the Mn(I) center to the nascent C–C bond in the transition state.

Across different solvents and metal systems, catalyst activation through CO loss always results in solvent binding within <5 ps. When experiments are performed in the presence of substrate for the reaction, then the solvent undergoes a slower (ns) substitution reaction by the substrate. Kinetic studies demonstrate that this is an overall second-order process (first-order in Mn complex and substrate), and for solvents, such as heptane and toluene, the second-order rate constant is close to the diffusion-controlled limit.^20,31^

## 5 Understanding Precatalyst Activation and Deactivation

**Figure 4:**
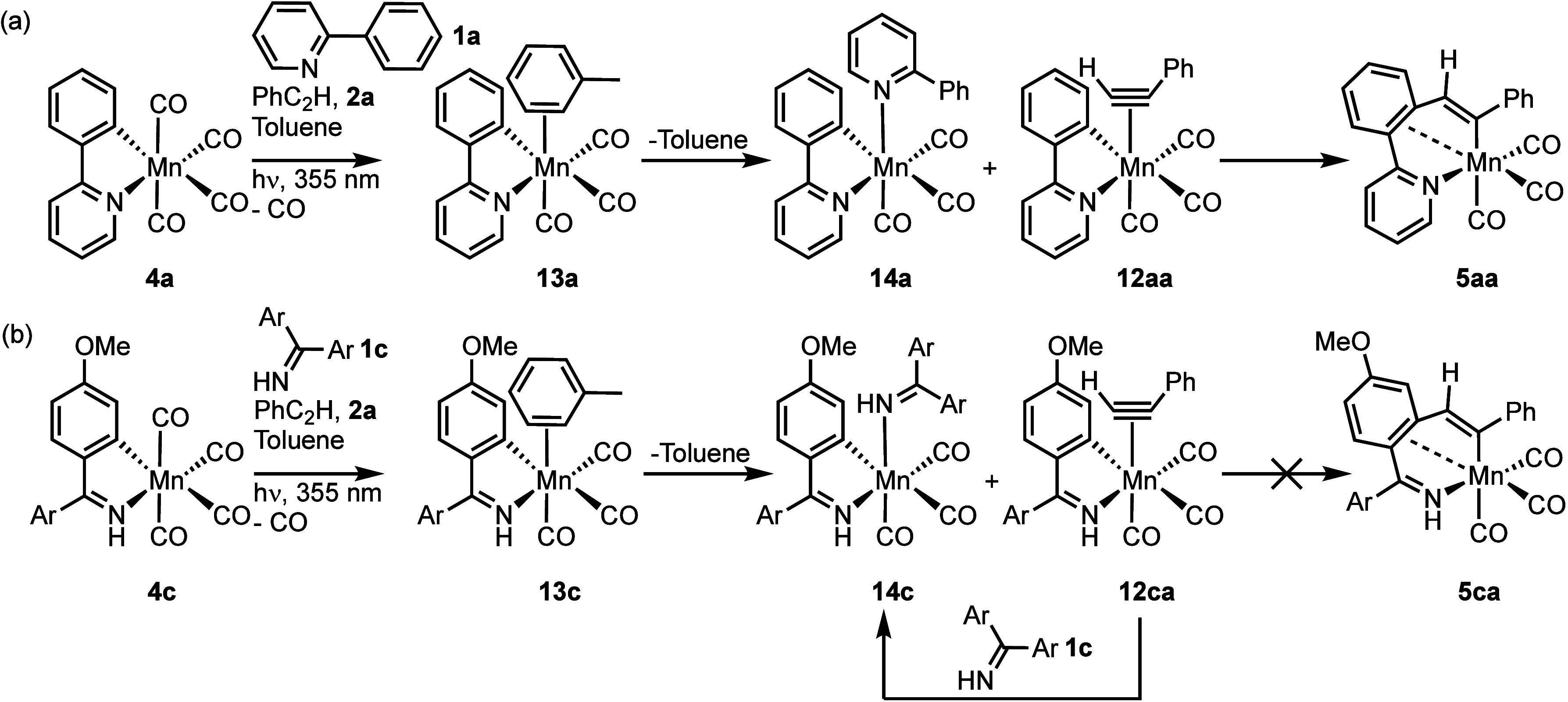
(a) Products formed from the photolysis of **4a** in the presence of **1a** and **2a** and (b) products formed from the photolysis of **4c** in the presence of **1c** and **2a** (Ar = C_6_H_4_-4-OMe).

The data described in Section 4 have provided important insight into the factors controlling the C–C bond formation steps during the alkenylation step of the catalytic cycle. The approach using TR^M^PS was also used to gain insight into how different substrates bind to precatalyst **4** following CO loss. Simulating the conditions used in catalytic reactions revealed a level of complexity in speciation. Experiments were performed using a toluene solution of **4a** containing both **1a** and **2a**.^32^ Competition between the two substrates for the manganese solvent complex occurred, which primarily depended on the relative concentration of **1a** and **2a**. A mixture of **12aa** and **14a** (Figure 4a) was observed; **12aa** then underwent the expected migratory insertion reaction to give **5aa**, and the N-bound complex **12a** remained unchanged. This indicated that under catalytic conditions a proportion of the catalyst was deactivated (at least on this time scale) by unproductive binding to the heterocycle **1a**. When imine-containing precatalyst **4c** was used in the presence of imine **1c** and PhC_2_H, then a mixture was also obtained: in this case, it consisted of **12ca** and **14c** (Figure 4b). However, in this instance, no migratory insertion reaction was observed; rather, substitution of the coordinated alkyne in **12ca** by **1c** occurred, and **14c** was the only product observed at longer time scale. This finding was linked to the considerably greater binding affinity of **1c** toward the metal when compared with **1a** but also demonstrated how catalyst speciation may be profoundly affected by the structural features of the substrate. Furthermore, these data demonstrate that solvent complexes play an important role in precatalyst activation and that the substitution of the weakly bound solvent by reaction substrate(s) is a key step. This is a fast (*k* ≈ 10^9^ mol^–1^ dm^3^ s^–1^ in heptane and ≈10^7^ mol^–1^ dm^3^ s^–1^ in toluene solution)^20^ second-order process and, although it is not clear if it proceeds through an interchange mechanism or a solvent dissociation–substrate addition pathway, it reinforces the important catalytic role of metal–solvent interactions.

**Figure 5:**
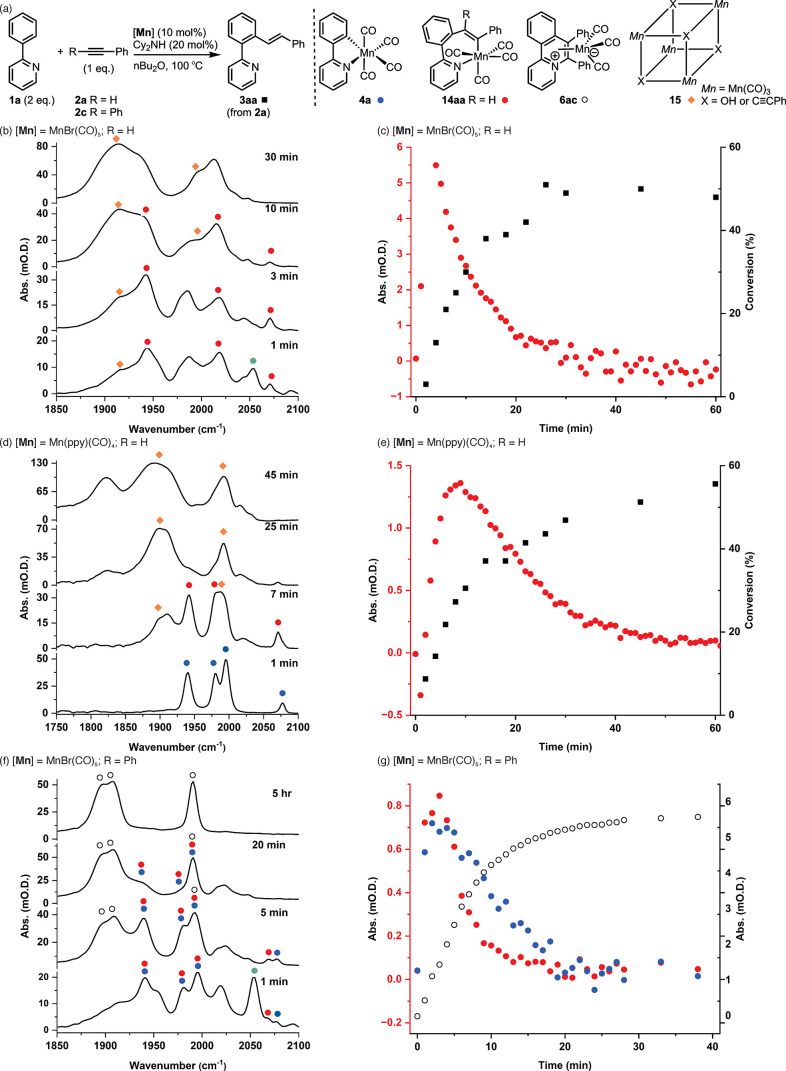
(a) Reaction scheme and key structures identified in the ReactIR study. (b) IR spectra recorded from the reaction between **1a** and **2a** catalyzed by MnBr(CO)_5_. (c) Kinetic profile showing the formation of **14aa** (red circles) and **3aa** (black squares). (d) IR spectra recorded from the reaction between **1a** and **2a** catalyzed by **4a**. (e) Kinetic profile showing the formation of **14aa** (red circles) and **3aa** (black squares). (f) IR spectra recorded from the reaction between **1a** and **2c** in the presence of [MnBr(CO)_5_]. (g) Kinetic profile showing the formation of **14ac** (red circles), **4a** (blue circles), and **6ac** (open circles).

Complementary studies were then undertaken to understand the process that underpins precatalyst activation and deactivation in thermal reactions. The latter occurs on a time scale of minutes and hours; therefore, studying the reaction by in situ IR spectroscopy proved an ideal method to investigate these phenomena (a Mettler-Toledo ReactIR instrumentation with a fixed Si probe was primarily used). The MnBr(CO)_5_-catalyzed alkenylation of 2-phenylpyridine by phenylacetylene was investigated first (Figure 5a).^3^ Under these conditions, the majority of MnBr(CO)_5_ had been consumed in 1 min (Figure 5b), and complex **14aa** arising from the formal migratory insertion of the alkyne into a Mn–C bond was the major product. Complex **14aa** was also observed when Mn(ppy)(CO)_4_ was used as a precatalyst (Figure 5d) and was formed over a slightly slower time scale than when MnBr(CO)_5_ was used (Figure 5c and 5e). This, coupled with the observation that when PhC_2_D was used as substrate the final product had only 78% D incorporation, lead to an alternative mechanistic proposal in which alkyne C–H bond activation is the first step in the activation of the Mn precatalyst. This would afford an alkynyl Mn(I) complex that, in turn, can activate 2-phenylpyridine through a σ-CAM reaction.^33,34^ Consistent with this proposal, an identical reaction using PhC_2_Ph, **2b**, resulted in the rapid formation of **6ab**, which showed that C–H bond activation of the 2-phenylpyridine was dominant in the absence of an acidic alkyne proton (Figure 5f and 5g).

These experiments provided important insight into the pathways leading to catalyst deactivation. Toward the end of the reaction, the spectra obtained by in operando IR monitoring of the reactions catalyzed by either [MnBr(CO)_5_] or [Mn(ppy)(CO)_4_] showed the presence of broad bands in the metal carbonyl region. These were demonstrated to belong to catalytically inactive hydroxide-bridged Mn–carbonyl clusters, **15**, when containing a {[Mn(CO)_3_(μ_3_-OH)]_*n*_}^35^ or {[Mn(CO)_3_(μ_3_-C≡CR)]_*n*_} building block. The formation of these hydroxide-bridged Mn clusters highlights the importance of the product-yielding protonation step in the reaction. There are a number of different potential acids in the system that can accomplish this step, including PhC_2_H, [NH_2_Cy_2_]^+^, 2-phenylpyridine, and water. DFT calculations demonstrated that protonation by either PhC_2_H or 2-phenylpyridine would be competitive; however, the barrier for protonation by water was higher. Therefore, at high substrate concentrations, the product is released by protonation by either substrate to restart the catalytic cycle. However, when the reaction is nearing completion and the substrate concentration is low, protonation by water dominates, which also results in the formation of the inactive Mn_*n*_ clusters (the catalyst dead end)

## 6 Evaluating the Role of Acid Additives in Mn-Catalyzed Reactions

**Figure 6:**
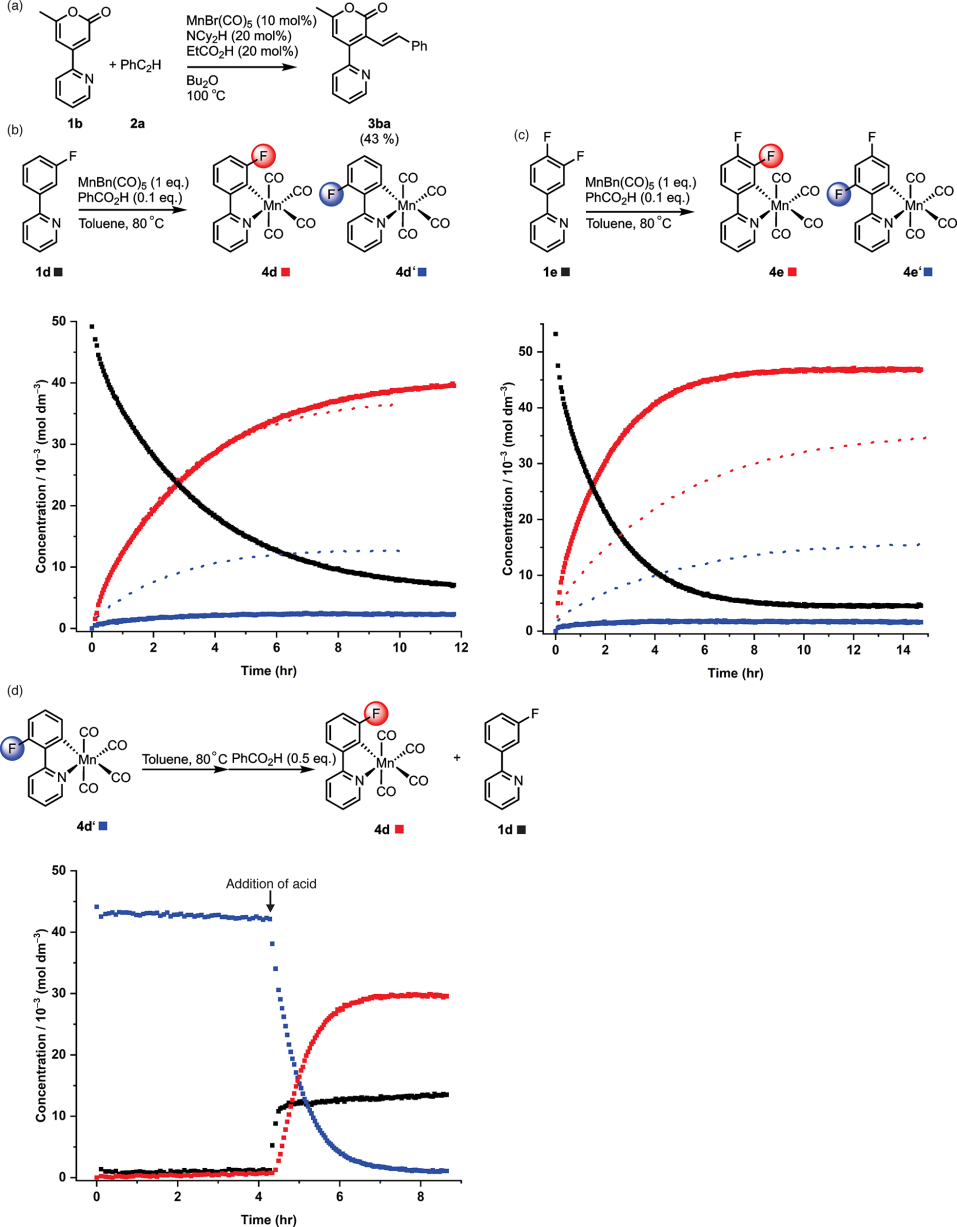
(a) Reaction between **1b** and **2a** to give **3ba**. (b) Top, reaction scheme showing products from the cyclomanganation of **1d**; bottom, kinetic profiles showing the loss of **1d** (black squares) and formation of **4d** (red) and **4d′**. Solid lines, experiment performed in the presence of PhCO_2_H; dashed line, experiment performed in the absence of PhCO_2_H. (c) Top, reaction scheme showing products from the cyclomanganation of **1e**; bottom, kinetic profiles showing the loss of **1e** (black squares) and formation of **4e** (red) and **4e′**. Solid lines, experiment performed in the presence of PhCO_2_H; dashed line, experiment performed in the absence of PhCO_2_H. (d) Effect of heating **4d′** and subsequent addition of PhCO_2_H showing the loss of **4d′** (blue) upon addition of acid and formation of **4d** (red) and **1d** (gray).

The formation of catalytically inactive Mn_*n*_ clusters, **15**, represents one of the key deactivation pathways for the catalyst system. The work described in Section 5 demonstrates that this occurs at low substrate concentrations when Mn–C bond protonation by water becomes competitive (at ∼60% conversion to product). It was hypothesized that all acidic species in a reaction may play a role in catalyst deactivation. With this in mind, the effects of ammonium salts, [NCy_2_H_2_]X, which would be generated in the initial C–H bond activation by the Mn and NCy_2_H, were studied.^36^ These experiments led to a number of important conclusions. First, the effect of adding salts [NCy_2_H_2_]X to the C–H bond functionalization of **1a** was highly substrate-dependent. When PhC_2_H was used as a coupling partner, little overall effect was observed, whereas for PhC(O)OC_2_H, a profound increase in yield was observed when the salts [NCy_2_H_2_]X were present compared with the reaction performed in the absence of additive or when NCy_2_H was used alone. When ethyl acrylate was used as substrate, the addition of the salts actually inhibited the reaction, and lower yields were observed. However, in operando IR studies demonstrated that the formation of Mn clusters was significantly retarded in the presence of [NCy_2_H_2_]X, which is consistent with alternative, nonwater-based pathways for Mn–C bond protonolysis. It was found that with the correct choice of acid additive, it was possible to perform alkenylation reactions that were not previously possible. For example, the alkenylated 2-pyrone could be generated in 43% yield when a mixture of NCy_2_H and EtCO_2_H was added to the reaction (Figure 6a).

The hypothesis underpinning these observations is that the acid additive promotes Mn–C bond cleavage to enable product liberation. Further evidence for this effect was provided through a study into the regiochemical outcome of the C–H bond activation in fluorinated 2-phenylpyridines.^21^ The influence of fluorine atoms in C–H bond activation reactions has been termed the “*ortho*-fluorine effect” because of the thermodynamic preference for a fluorine atom to occupy a position ortho to the C–H bond that is activated (a contribution from the M–C bond strength is key).^37−40^ In the absence of an acid additive, the cyclomanganation of a range of fluorinated 2-phenylpyridines was shown to be kinetically controlled with two isomers formed (Figure 6b,c). Addition of acid to the reaction resulted in a change in the regioselectivity, and the product with an *ortho*-fluorine atom was obtained. Furthermore, addition of acid to the less thermodynamically favorable isomer of complex **4d′** resulted in isomerization to the thermodynamically preferred product, **4d** (Figure 6d). These data illustrate how acid additives enable Mn–C bond cleavage, along with a thermodynamic distribution of products.

## 7 First Direct Experimental Evidence for the Concerted Metalation Deprotonation Pathway

The experiments described in Section 6 illustrate the key role played by acid additives in Mn-catalyzed reactions. A survey of Mn-catalyzed reactions illustrates that the addition of carboxylic acids or their conjugate bases may have a positive influence on the outcome of the reactions. This reflects the more general observation that carboxylate or carbonate additives may enhance the rate of C–H bonding functionalization reactions.^41^ This is one of the most important synthetic findings in recent years and has underpinned many advances in metal-mediated C–H bond functionalization reactions. Detailed computational studies have shown that this effect may be linked to an intramolecular C–H bond deprotonation event involving the coordinated substrate and additive, which has been referred to as concerted metalation deprotonation (CMD)^42,43^ or ambiphilic metal ligand activation (AMLA-6 in this example).^44−46^ Despite the compelling computational evidence for this mechanistic pathway, the key intermediates in this process have not been observed, and its mechanistic role has relied in many cases on secondary experimental evidence from kinetic isotope measurements.^47^

Preliminary DFT calculations on the Mn–carbonyl system indicated that protonation of a Mn–C bond might be observed experimentally, whereas the microscopic reverse (C–H bond activation by a coordinated acetate) is thermodynamically unfavorable.^4^ However, proton transfer reactions are expected to be rapid, and the calculated barriers for this step in CMD reactions have been shown to be low. An experiment was designed in which irradiation of **4a** in neat acetic acid would be performed. This would ensure that the binding of the acetic acid to the metal and subsequent proton transfer events would occur rapidly and would not be obscured compared with a slower solvation step.

**Figure 7:**
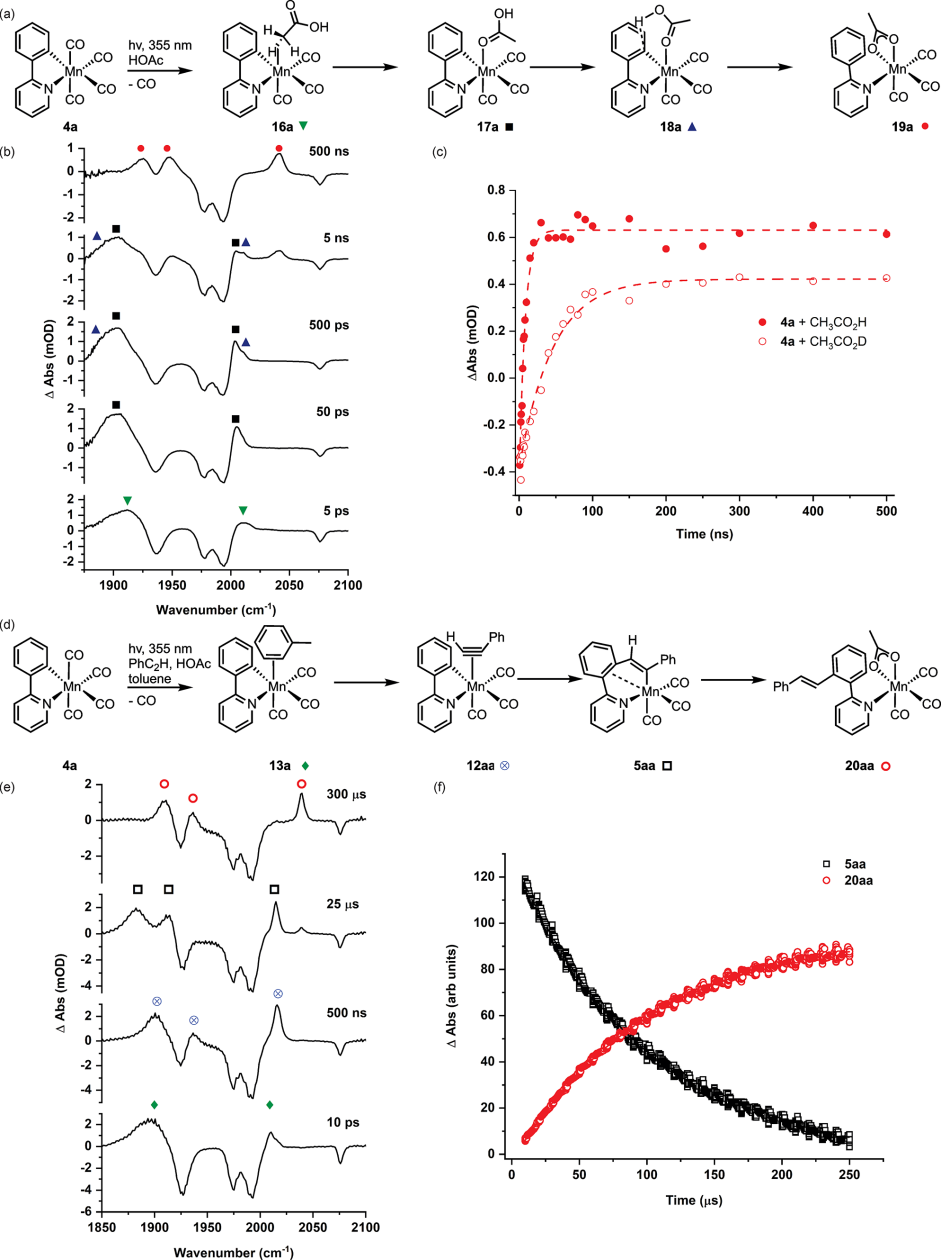
(a) Reaction scheme showing products formed from the photolysis of **4a** in the presence of CH_3_CO_2_H. (b) TRIR spectra for the photolysis of **4a** in the presence of CH_3_CO_2_H. (c) Kinetic profile for the formation of **19a** from the reaction with CH_3_CO_2_H and CH_3_CO_2_D. (d) Reaction scheme showing products formed from photolysis of **4a** in the presence of PhC_2_H and CH_3_CO_2_H. (e) TRIR spectra for the photolysis of **4a** in the presence of PhC_2_H and CH_3_CO_2_H. (f) Kinetic profile for the formation of **20aa** from **5aa**.

Photolysis of an acetic acid solution of **4a** resulted in ultrafast CO dissociation and the observation of several different species (Figure 7a). At very short pump–probe delays, binding appeared to primarily occur to the methyl group of the acetic acid, **16a**, prior to isomerization of the oxygen-bound form, **17a**. On the basis of the change in IR band positions (Figure 7b), another isomerization was proposed where the acid is engaged in hydrogen bonding to 2-phenylpyridine, **18a**, such a structure had been predicted by DFT calculations^26,46^ as a minimum, and our experiments provided direct experimental evidence for its existence. Finally, a proton transfer to give **19a** was observed—this step exhibited a large kinetic isotope effect (5.8 ± 1.0) (Figure 7c)—consistent with O–H/O–D bond cleavage. The experiment was repeated with different manganacycles and carboxylic acids, and although the rate constants for each of the steps showed some variation, the general pattern and kinetic isotope effect (KIE) were present in each case. Notably, performing the experiment in toluene solution resulted in the formation of the same complex, **19a**, but none of the previously observed intermediates were present, and a much lower KIE was obtained. This was due to the rate of solvent substitution by HOAc being slow compared with the rate of protonation. This highlights the power of being able to perform experiments in a neat substrate because fast mechanistic events may otherwise be obscured by slower steps.

Crucially, the temporal range provided by TR^M^PS was demonstrated by performing an experiment in a toluene solution of **4a**, PhC_2_H, and HOAc. It was possible to observe the initial solvation of the light-activated complex to give **13a** on a ps time scale, solvent substitution by the alkyne to give **12aa** on a ns time scale, migratory insertion of the alkyne into the Mn–C bond to give **5aa** over 20 μs, and then over the course of 250 μs, protonation of the manganacycle to give **20a** in which the product of the overall catalytic reaction was bound to the metal (Figure 7d–f). Therefore, catalytic events covering nearly 8 orders of magnitude of time were observed in a single experiment.

## 8 Outlook

Linking experiments on the fastest time scale (ps−μs) with those monitoring occurring over seconds and hours can provide unique insight into the cradle-to-grave behavior of a catalyst system. By initially starting with traditional methods for the examination of mechanisms involving Mn(I) carbonyl species, particularly NMR and MS, we recognized the potential of IR (from fast to slow time scales) as being critical in understanding several key processes and reaction steps. The experimental and computational evidence support a catalytic cycle for C–H bond functionalization based solely on Mn(I) intermediates with a core fac-Mn(CO)_3_ framework. Upon catalyst deactivation, some of higher-order clusters have Mn in a higher oxidation state,^35^ and there is evidence that Mn(II) compounds are formed in the [MnBr(CO)_5_]-mediated hydrogenation of *N*-heterocycles.^48^ However, the strong ligand field provided by the fac-Mn(CO)_3_ structural unit ensures that the resulting low-spin d^6^ electronic configuration is robust to both oxidation and reduction and also promotes the binding of π-acceptor ligands (e.g., alkenes and alkynes) to the metal. It is this factor that presumably enables Mn–carbonyl complexes to so effectively mimic the behavior of 4d and 5d metal complexes.

The TR^M^PS technique was particularly suited to the study of these Mn(I) carbonyls, which take advantage of the photochemical generation of a nonequilibrium state species that can interact with various ligands, substrates, and additives, thereby shining light on molecular events, such as solvent binding and dissociation, substrate binding, migratory insertion, and protonation. Indeed, the observation that after ligand loss the metal initially coordinates any and all components of the reaction mixture in a stochastic fashion should be an important consideration in all catalytic cycles. All of these processes sit as pillars within Mn(I)-catalyzed C–H bond functionalization reactions and can, moreover, be exploited in other photoactivatable systems.^49^ We recognize that there is significant potential in how our approach can be exploited and developed in new systems, which we would like the wider catalysis community to consider.

## Abbreviations

TR^M^PS: time-resolved multiple probe spectroscopy
IR: infrared
KIE: kinetic isotope effect
Tol: 4-tolyl
2-pym: 2-pyrimidyl
2-py: 2-pyridyl
MI: migratory insertion
